# The Contribution of Grammar, Vocabulary and Theory of Mind in Pragmatic Language Competence in Children with Autistic Spectrum Disorders

**DOI:** 10.3389/fpsyg.2017.00996

**Published:** 2017-06-15

**Authors:** Clara Andrés-Roqueta, Napoleon Katsos

**Affiliations:** ^1^Department of Developmental, Educational Social and Methodological Psychology, Universitat Jaume I de CastellóCastellón, Spain; ^2^Department of Theoretical and Applied Linguistics, University of CambridgeCambridge, United Kingdom

**Keywords:** pragmatics, theory of mind, vocabulary, grammar, autistic spectrum disorders (ASD), structural language, social cognition

## Pragmatic competence in children with ASD and other developmental disorders

### Pragmatic competence in ASD

Pragmatic skills enable children to produce and comprehend words and sentences in ways that are appropriate to the conversational context. While structural language is known to vary widely in children with Autistic Spectrum Disorders (ASD), pragmatic language has been claimed to be consistently impaired within this population, and has been considered a hallmark of ASD (Volden and Phillips, 2010). Specially, people with ASD frequently demonstrate unusual or inappropriate conversational behavior and deficits in a wide range of pragmatic skills (Philofsky et al., 2007). These difficulties have been experimentally demonstrated in detecting violations of maxims of conversation (Surian et al., 1996), understanding figurative language (Happé, 1993; Norbury, 2005), using context to disambiguate polysemous words (Jolliffe and Baron-Cohen, 1999; Brock et al., 2008), managing topic maintenance and topic shifts (Volden and Phillips, 2010), and comprehending humor, drawing inferences from narratives and understanding indirect requests (Ozonoff and Miller, 1996).

Pragmatic difficulties are often attributed to intrinsic features of ASD. These include a weaker tendency to integrate information from the context (Weak Central Coherence; Happé and Frith, 2006), a deficit in Theory-of-Mind (ToM) that prevents children with ASD from inferring intentions and mental states of other people (Baron-Cohen et al., 1985), a deficit in executive functions such as poor inhibition or cognitive flexibility (Hill, 2004) or lack of social motivation as a result of an attenuated social instinct (Chevallier et al., 2012).

### Pragmatics in other communication disorders

Children with developmental disorders with communication problems in the absence of ToM deficits like Specific Language Impairment (SLI), also display pragmatic difficulties when screening instruments and conversational analysis are used (Adams, 2002; Norbury et al., 2004). Experimental studies have shown that children with SLI face deficits in sensitivity to maxims of conversation, figurative language understanding, narrative or use of context to resolve ambiguities (Surian et al., 1996; Norbury, 2004, 2005; Brock et al., 2008; Katsos et al., 2011; Norbury et al., 2014), and that their pragmatic skills are in keeping with levels of their structural language, as they perform as successfully as younger typically-developing (TD) children matched on language level at experimental tasks.

Nevertheless, the extent of pragmatic impairments in children with ASD and other children with social communication disorders, as well as the underlying cause of these impairments, is still an open question for research and practitioners (Adams, 2002; Norbury, 2014).

## Potential causes of pragmatic difficulties in ASD

Recent research has questioned the traditional views of pragmatic competence in ASD: Are children with ASD universally challenged by pragmatics? Are these challenges due to some deficit of ToM intrinsic to ASD?

### The role of ToM

The literature reveals reliable associations between the process of understanding the ironic meaning of utterances and ToM skills. For example, there is correlational evidence between success with irony understanding and passing False Belief tasks both in children with ASD and TD (Happé, 1993; Filippova and Astington, 2008), as well as evidence that irony comprehension and ToM processing activate the same neural regions in neuro-typical adults (Spotorno et al., 2012).

However, Norbury (2004, 2005, 2014) and Happé (1993) reached different conclusions as regards the role of ToM in pragmatic competence of children with ASD. An alternative proposal is that the pragmatic language deficits observed in children with ASD are due to difficulties with grammar and vocabulary, known collectively as structural language (Norbury, 2005; Gernsbacher and Pripas-Kapit, 2012).

### The role of structural language: grammar and vocabulary

In particular, Norbury (2004, 2005) put this hypothesis to the test by studying four groups of children with the presence or absence of ASD and of Language Impairment (LI) in metaphor and idiom tasks (ASD-LI; ASD+LI; LI; and age-matched TD). Crucially, structural language competence was measured both expressively and receptively, and both in terms of vocabulary and grammar. It was found that all groups with language impairment (ASD+LI and LI) were indeed impaired in comprehension of metaphors and idioms, but the group with ASD-LI (without LI) performed as well as the TD participants. Moreover, regression analyses revealed that structural language and world-knowledge were the critical predictors for idioms and metaphors, whereas ToM was not. Furthermore, a recent meta-analytic review of experimental studies in figurative language concluded that the differences between children with ASD and TD groups were not statistically significant when the groups were matched on language ability (Kalandadze et al., 2016).

Likewise, it has been demonstrated that children with ASD perform as well as TD peers on the ability to detect pragmatic violations, such as utterances that are literally true but pragmatically under-informative (e.g., “*some of the apples are inside the boxes*” when shown a picture where *all* of the apples are inside the boxes), and that higher verbal IQ scores predicted higher sensitivity to under-informativeness within the ASD group (Chevallier et al., 2010). Similar conclusions are reached in studies with an adult ASD population (Pijnacker et al., 2009). However, in the two last studies, the lack of ToM measures prevents establishing a unique contribution of structural language.

## Two different pragmatic skills: linguistic- vs. social-pragmatics

Here we propose that the relationship between pragmatics, structural language and ToM is not “fixed” but rather modulated by specific properties of the interaction (lexical, syntactic and social-interactional aspects)^1^. In addition to differences in measurement of independent factors (e.g., measuring ToM and structural language in different ways), an element that may explain the variation in research findings within the ASD population is that different ways of testing pragmatics may differentially engage structural language and ToM.

A distinction between types of pragmatic inferences has gained much support in the theoretical pragmatics literature, classified by the extent to which they require ToM skills: Sperber (1994) mentions “Egocentric Relevance” (which does not involve ToM skills), “Allocentric Relevance” (which requires 1st order ToM) and “Gricean” interpretative strategies (which require 2nd order ToM); Levinson (2000) distinguishes between “generalized” and “particularized” pragmatic inferences; Recanati (2004) introduces a distinction between “primary” and “secondary pragmatic processes”; and more recently, O'Neill (2012) uses the terms “social pragmatics,” “mindful pragmatics” and “cognitive pragmatics” while Kissine (2012) discusses inter-subjective and non-intersubjective aspects of language use. This view has also been supported by empirical research in children with and without communication disorders, suggesting that, for some kinds of pragmatics, a sentence may be fully interpretable based on pragmatic norms and the context as provided from the listeners' egocentric point of view, without the need to infer the speakers' mental state (de Villiers et al., 2007; Kissine, 2012; O'Neill, 2012; Kissine et al., 2015; Janke and Perovic, 2016).

At this point, we suggest two new terms: linguistic-pragmatics and social-pragmatics. We think that they are more intuitively transparent as regards the role of structural language and ToM in each type of pragmatic skill. The term *linguistic-pragmatics* would be for those cases of pragmatics where structural language and competence with pragmatic norms are enough to perform successfully in the task, while we use the term *social-pragmatics* for those circumstances where in addition to structural language and pragmatics, the child needs competence with ToM, and specifically the ability to represent other people's intentions, desires and beliefs.

### Linguistic-pragmatics

Sensitivity to informativeness, as tested by Chevallier et al. (2010), Katsos et al. (2011), and Pijnacker et al. (2009) is a case in point of “linguistic-pragmatics.” For example, in order to reject pragmatically infelicitous sentences of a speaker saying that “*some of the apples are inside the boxes*” (given a picture in which *all* of the apples are inside the boxes), a child need to draw on vocabulary knowledge (a child who has mastered the semantic meaning of “some” and “all” will know that “all” is a more informative expression), together with sensitivity to the pragmatic maxim that instructs speakers to avoid being under-informative. However, demands on ToM are minimal, because the knowledge that is necessary to evaluate if the utterance is informative or not is visually accessible and shared between the child and the speaker.

As a result, empirical evidence shows that structural language is the key predictor for success with informativeness (Pijnacker et al., 2009; Chevallier et al., 2010 in participants with ASD; Katsos et al., 2011, in participants with SLI).

### Social pragmatics

In contrast, there are cases that do require using ToM skills. The irony task used by Happé (1993) is a case in point.

In one of the stories, the main character (David) is baking a cake and places the eggs in the batter without removing the shells, and his dad says: “*What a clever boy you are, David!*.” Here, in order to understand this ironic utterance, a child needs to use his/her competence with structural language to grasp the literal meaning, together with the pragmatic maxim that enjoins interlocutors to be relevant and truthful. Moreover, the child does need to use ToM skills for two reasons. First, in order to avoid attributing to David's dad a false belief (David is clever), that would nevertheless be consistent with the literal meaning of the utterance. And second, in order to take into account the true belief of David's dad (David is not clever) that is inconsistent with the literal meaning of the sentence, but becomes consistent once pragmatic inference has taken place.

Consequently, the *Strange Stories* task (Happé, 1994), which is constructed on similar principles as Happé's (1993) irony task, could be a good measure for social-pragmatic skills. Although this task has been typically used to assess mentalizing through the recognition of the communicative intentions of people using indirect or non-literal utterances, the characters of the stories have *unusual* or *unexpected* mental states that are not compatible with the literal meaning of what they say. Correctly inferring those mental states is a prerequisite for making the pragmatic inferences that allow the participant to tell if what the characters say is appropriate or not for the context.

We should clarify here that we do not visualize the distinction between linguistic-pragmatics and social-pragmatics as one to do with pragmatic phenomena *per se*, but with the communicative situation. Tasks that measure informativeness, for example, need not always fall under the umbrella of linguistic-pragmatics. There may well be cases where sensitivity to informativeness will require ToM and therefore be considered social-pragmatic, e.g., in cases where the speaker but not the hearer has only partial knowledge of the facts.

## Theoretical, experimental, and clinical implications

The different roles of ToM and structural language in pragmatics tasks may help to explain some of the variance in findings from previous reports on children with ASD. We expect that the linguistic-pragmatic difficulties of children with ASD (and of children with other developmental disorders like SLI), will be in keeping with structural language (grammar and vocabulary) in tasks such as sensitivity to under-informativeness, but in keeping with their ToM skills in tasks that require social-pragmatic competence, such as irony stories from Happé (1994) Strange Stories task.

Structural language is implicated in the success with pragmatics, including metaphor understanding (Norbury, 2004), informativeness (Pijnacker et al., 2009; Chevallier et al., 2010), idioms (Norbury, 2005), use of context for disambiguation (Brock et al., 2008), and it is one of the significant (if not the only) predictors of success. This highlights the importance of considering structural language when assessing pragmatic difficulties, in order to establish whether any pragmatic difficulties go beyond the overall linguistic differences that a child presents.

Furthermore, structural language must have a key role in intervention. It is likely that in addition to interventions that directly target pragmatic competence, support for structural language is the one component that will benefit all children who show pragmatic difficulties (Kalandadze et al., 2016). Additionally, intervention with ToM is also likely to support pragmatic language in some specific situations.

Finally, the distinction between linguistic- and social-pragmatics may help clarify for some questions pertaining to diagnostic categories. Social (Pragmatic) Communication Disorder has recently been proposed as a distinct diagnostic category (see American Psychiatric Association, 2013). Among others, this disorder includes deficits in using communication for social exchange, adapting communication style to the context, following rules of conversation or narrative convention and understanding implicit or ambiguous language (Norbury, 2014). If our proposal is correct, these deficits are at least partially distinct, as they include both what we called linguistic-pragmatic and social-pragmatic competences. They are also likely to be present in children with ASD, SLI and other disorders, depending on the extent of structural language and ToM impairments.

Screening instruments and diagnostic procedures that measure communicative and pragmatic competence may also take into account the distinction between linguistic- and social-pragmatic competences, which at present tend not to be differentiated (e.g., in the Children's Communication Checklist–2, CCC-2, Norbury et al., 2014).

## Author contributions

Both authors (CA and NK) have contributed in the selection, reflective, and analytical reading of the different studies, making a thorough comparison of the methods used, and discussing the reflections between both to translate it into the manuscript. Moreover, every section has been written and discussed equally by CA and NK, so the opinion given in the ms is from both authors. As the first author, CA has been responsible for minor issues in relation to the final wording of the document, but both authors have revised together the final manuscript before sending it.

### Conflict of interest statement

The authors declare that the research was conducted in the absence of any commercial or financial relationships that could be construed as a potential conflict of interest.

## Footnotes

^1^An additional interaction between these concepts arises if participants use their mastery of the syntax of complementation to pass False Belief tasks (de Villiers et al., 2003). This is a possibility that we do not explore here but should be taken into account in future work.
